# Integrating US-guided FNAB, BRAF^V600E^ mutation, and clinicopathologic characteristics to predict cervical central lymph-node metastasis in preoperative patients with cN0 papillary thyroid carcinoma

**DOI:** 10.1007/s00405-023-08156-w

**Published:** 2023-08-04

**Authors:** Ning Ma, Hai-Ying Tian, Zhao-Yan Yu, Xin Zhu, Dai-Wei Zhao

**Affiliations:** 1 Clinical Medical College, Guizhou Medical University, Guiyang, China; 2 Department of Vascular and Thyroid Surgery, Guizhou Provincial People’s Hospital, Guiyang, China; 3 Department of Ultrasound, Guizhou Provincial People’s Hospital, Guiyang, China; 4 Department of Thyroid and Breast Surgery, Second People’s Hospital of Guizhou Province, No. 206, South Section of Xintian. Avenue, Guiyang, 550004 China; 5 Department of Breast and Thyroid Surgery, Guiqian International General Hospital, No. 1 Dongfeng Avenue, Wudang District, Guiyang, 550024 China

**Keywords:** Fine-needle aspirations biopsy, Papillary thyroid carcinoma, Central lymph-node metastasis, BRAF^V600E^ mutation, Prophylactic central lymph-node dissection, Clinically negative central compartment lymph node

## Abstract

**Background:**

The prevalence of cervical central lymph-node metastasis (CLNM) is high in patients with papillary thyroid carcinoma (PTC). There is considerable controversy surrounding the benefits of prophylactic central lymph-node dissection (pCLND) in patients with clinically negative central compartment lymph nodes (cN0). Therefore, it is crucial to accurately predict the likelihood of cervical CLNM before surgery to make informed surgical decisions.

**Methods:**

Date from 214 PTC patients (cN0) who underwent partial or total thyroidectomy and pCLND at the Guizhou Provincial People's Hospital were collected and retrospectively analyzed. They were divided into two groups in accordance with cervical CLNM or not. Their information, including clinical characteristics, ultrasound (US) features, pathological results of fine-needle aspirations biopsy (FNAB), and other characteristics of the groups, was analyzed and compared using univariate and multivariate logistic regression analyses.

**Results:**

A total of 214 patients were eligible in this study. Among them, 43.5% (93/214) of PTC patients had cervical CLNM, and 56.5% (121/214) did not. The two groups were compared using a univariate analyses, and there were no significant differences between the two groups in aspect ratio, boundary, morphology, component, and BRAF^V600E^ (*P > *0.05), and there were significant differences between gender, age, maximum tumor size, tumor location, capsule contact, microcalcifications, color Doppler flow imaging (CDFI), and Hashimoto's thyroiditis (HT) (*P < *0.05). A multivariate logistic regression analysis was performed to further clarify the correlation of these indices. However, only age (OR = 2.455, *P = *0.009), maximum tumor size (OR = 2.586, *P = *0.010), capsule contact (OR = 3.208, *P = *0.001), and CDFI (OR = 2.225, *P = *0.022) were independent predictors of cervical CLNM. Combining these four factors, the area under the receiver-operating characteristic (ROC) curve for the joint diagnosis is 0.8160 (95% 0.7596–0.8725). Univariate analysis indicated that capsule contact (*P* = 0.001) was a possible predictive factor of BRAF^V600E^ mutation.

**Conclusions:**

In conclusion, four independent predictors of cervical CLNM, including age < 45 years, tumor size > 1.0 cm, capsule contact, and rich blood flow, were screened out. Therefore, a comprehensive assessment of these risk factors should be conducted when designing individualized treatment regimens for PTC patients.

## Introduction

In recent years, the incidence of thyroid cancer has substantially increased worldwide [1, 2]. The most common subtype is papillary thyroid carcinoma (PTC), accounting for almost 90% of all thyroid malignancies. Despite a rising incidence, the mortality rate from thyroid cancer has not changed significantly over the past 5 decades, which is in part related to well-developed treatment procedures [3]. Although PTC has a good prognosis, cervical central lymph-node (CLN) occult metastasis rate of patients with clinically negative central compartment lymph node (cN0) could be as high as 84.3%, which changed tumor stage and postoperative management [4]. Owing to anatomical factors including the carotid pulse and the potential for small metastases not detectable via imaging examination to be present, predicting central lymph-node metastasis (CLNM) preoperatively can be challenging. While, CLNM is generally considered a risk factor for poor prognosis in PTC, so the status of lymph node (LN) is also an important basis for judging recurrence and LN dissection [5]. At the same time, for patients with CLNM of PTC, the operation caused by persistent LNs’ recurrence will increase the risk of postoperative complications such as dyspnea, asphyxia, hypoparathyroidism, etc. [6]. Thus, it is necessary to predict CLNM in cN0 PTC patients to determine whether prophylactic central lymph-node dissection (pCLND) is needed. However, the use of pCLND in all cN0 PTC patients is still controversial [7]. Prophylactic CLND can improve disease-free survival and decrease the local recurrence rate, but it also increases the rate of surgical complication, such as recurrent laryngeal nerve injury and permanent hypoparathyroidism [6]. Therefore, routine pCLND poses additional surgical risks, and it is crucial to screen PTC patients for predictors of cervical CLNM [8].

Currently, high-resolution ultrasound (US) is recommended as the initial auxiliary approach for distinguishing between benign and malignant thyroid nodules. The combination of routine health assessments and US-guided fine-needle aspiration biopsy (FNAB) has significantly increased the rates of PTC detection and preoperative diagnosis. However, US has lower diagnostic efficiency in detecting CLNM than in detecting lateral cervical lymph node (LCLN) [9].

Several genetic mutations have been linked to the molecular pathogenesis of PTC, and examining these genes can aid in the diagnosis and prognostic evaluation of patients with PTC. The BRAF^V600E^ mutation is the most common genetic alteration in thyroid cancer, with an incidence ranging from approximately 30% to more than 80% [10]. This mutation has been linked to poor clinicopathological outcomes of PTC, such as LNM, disease recurrence, and patient mortality [11].

Although controversial, pCLND is still recommended in many countries [12]. Our study aimed to identify important predictors from medical information for pCLND. We retrospectively reviewed 214 consecutive PTC patients who experienced thyroidectomy plus pCLND. Univariate and multivariate analyses were also conducted to estimate the relationship between cervical CLNM and BRAF^V600E^ mutation status, clinical characteristics, preoperative US features, and postoperative clinicopathological features of cN0 stage PTC patients, with the goal of identifying predictors of CLNM.

## Materials and methods

### Patient selection

Consecutive inpatients who had US-guided FNAB reporting suspicious PTC or PTC and underwent partial or total thyroidectomy and pCLND from October 2021 to May 2023 in the Department of Vascular and Thyroid Surgery, Guizhou Provincial People's Hospital, Guiyang, China. After surgery, all patients were divided into two groups, pathologically CLNM positive (group A) and pathologically CLNM negative (group B). The clinical characteristics, US features, and pathological FNAB results were retrospectively reviewed and analyzed. Data about the patients were extracted from the medical records. The inclusion criteria included: (1) histologically confirmed PTC; (2) detection of BRAF^V600E^ mutation for thyroid lesions with specimens from preoperative FNAB; (3) PTC with or without cervical CLNM should be confirmed by pathological specimens. The exclusion criteria included: (1) patients who underwent thyroid nodule minimally invasive ablation, any cervical surgery, chemical therapy, or radiotherapy; (2) imaging suspicious or FNAB confirmed LCLN metastasis; (3) US imaging date were incomplete; (4) thyroid nodules were pathologically confirmed as other kinds of thyroid carcinomas. Based on the inclusion and exclusion criteria, all 214 patients included in this study were diagnosed with PTC by FNAB and BRAF^V600E^ mutation analysis prior to surgery.

### Conventional and color doppler ultrasound

Prior to FNAB, nodules underwent color doppler US examination using the SuperSonic Imagine of Aixplorer Sxc6-1 (4–15 MHz) and SonoScape 12L-A (3–17 MHz) line array probes to assess various US features, including nodule size, tumor location, boundary, sharpness, internal echo, aspect ratio, capsular contact, and microcalcifications. Following conventional grayscale US, color Doppler flow imaging (CDFI) was employed to identify the presence or absence of perinodular and intramodular vascular distributions.

### US-guided FNAB

**Fig. 1:**
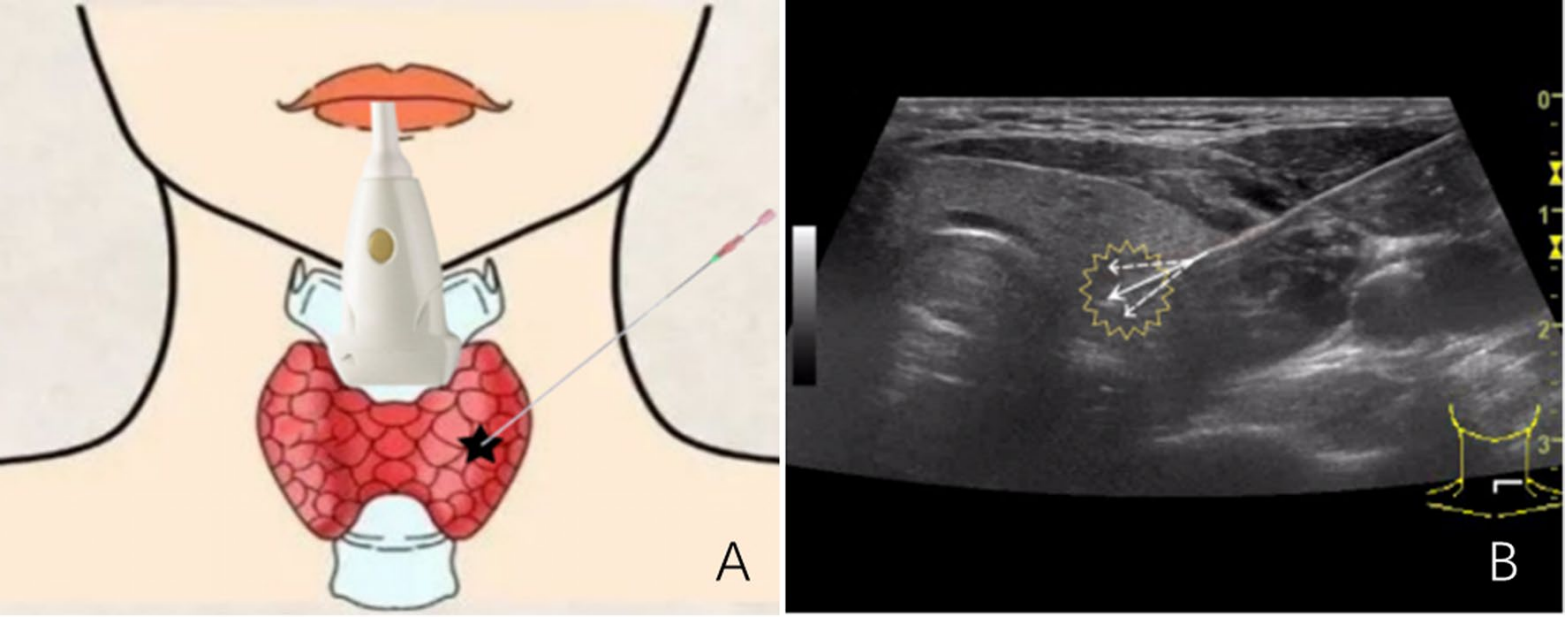
**A** Schematic diagram of ultrasound (US)-guided fine-needle aspirations biopsy (FNAB) (thyroid nodule). **B** US image of multipoint and multidirection US-guided FNAB (thyroid nodule). (The white solid line arrow indicates the puncture needle, and the white dashed line arrow indicates multipoint and multidirectional puncture)

The described procedure involves the use of US to guide the insertion of fine needles into a suspect node to obtain tissue samples for further analysis. The patients were positioned supine with their backs raised and heads tilted back to facilitate the procedure. The needles were inserted at multiple points and angles to ensure that sufficient tissue was obtained (Fig. 1). The obtained specimen was visually evaluated for suitability before being sent for cytological examination and BRAF^V600E^ mutation analysis. FNAB specimens should be read by an experienced cytopathologist and be reported according to the Bethesda Classification System [13]. The procedure appears to be a minimally invasive and efficient method for obtaining tissue samples for diagnostic purposes.

### ***BRAF***^***V600E***^*** mutation analysis***

BRAF^V600E^ mutation analysis was performed at the pathology department of our hospital. Briefly, DNA was extracted from the FNAB specimens and real-time polymerase chain reaction (PCR) was performed using the Stratagene Mx3000P real-time QPCR system (Agilent, LA Jolla, California, USA). The gene encoding BRAF^V600E^ was amplified with specific primers under the following thermal cycling conditions: an initial denaturation of 1 cycle for 5 min at 95 °C, followed by 15 cycles of 95 °C for 25 s, 64 °C for 20 s, and 72 °C for 20 s with a final step of annealing and elongation of 31 cycles at 93 °C for 25 s, 60 °C for 35 s, and 72 °C for 20 s. The BRAF^V600E^ mutation status of each primary PTC was determined using the Human BRAF Gene V600E Mutation Fluorescence PCR Diagnostic Kit (Amoy Aide Diagnostics). The FAM signals of the mutation detection system indicate the mutation status of the sample. The HEX/VIC signals were used to assess the internal control status. The FAM Ct value was evaluated for each sample: samples with an FAM Ct value ≥ 27 were classified as negative or undetected, while samples with a FAM Ct value < 27 were classified as positive for the mutation. The run files were interpreted according to the manufacturer's instructions.

### Thyroid surgery and pathologic analysis

In cases of unilateral thyroid tumors, the medical team performed unilateral thyroidectomy and isthmectomy. Meanwhile, total thyroidectomy was conducted when there were bilateral tumors or signs of extrathyroidal extension during intraoperative examination. Ipsilateral pCLND was carried out in cases of unilateral tumors, while bilateral pCLND was performed in patients with bilateral tumors. The pCLND boundary was established by the carotid arteries laterally, hyoid bone superiorly, and suprasternal notch inferiorly, including pre- and paratracheal nodes, prelaryngeal lymph nodes, perithyroidal nodes, and lymph nodes along recurrent laryngeal nerves. During the procedure, the medical team ensured the protection of the parathyroid gland and recurrent laryngeal nerve. The postoperative specimens (thyroid tissue and central cervical lymph-nodes) were sent for paraffin sections and finally confirmed as PTC with or without CLNM by experienced pathologists.

### Statistical analysis

The statistical analysis was carried out in SPSS ver. 25.0 (SPSS Chicago, IL, USA). Data were presented as mean ± standard deviation. Comparison of continuous and categorical variables was analyzed by Pearson chi-square test or Fisher’s exact test, respectively. Univariate analysis was used to examine the relation between predicting elements and cervical CLNM or BRAF^V600E^ mutation. Additionally, multivariate logistic regression was performed to assess independent risk factors for cervical CLNM, using the factors screened by univariate analysis. Receiver-operating characteristic (ROC) curves of the statistically significant factors and the predictive equation were plotted to analyze the reliability of the results of multivariate analysis. All *P* values were two-sided, with a value < 0.05 being treated as significant.

## Results

### Clinical and US characteristics

**Fig. 2:**
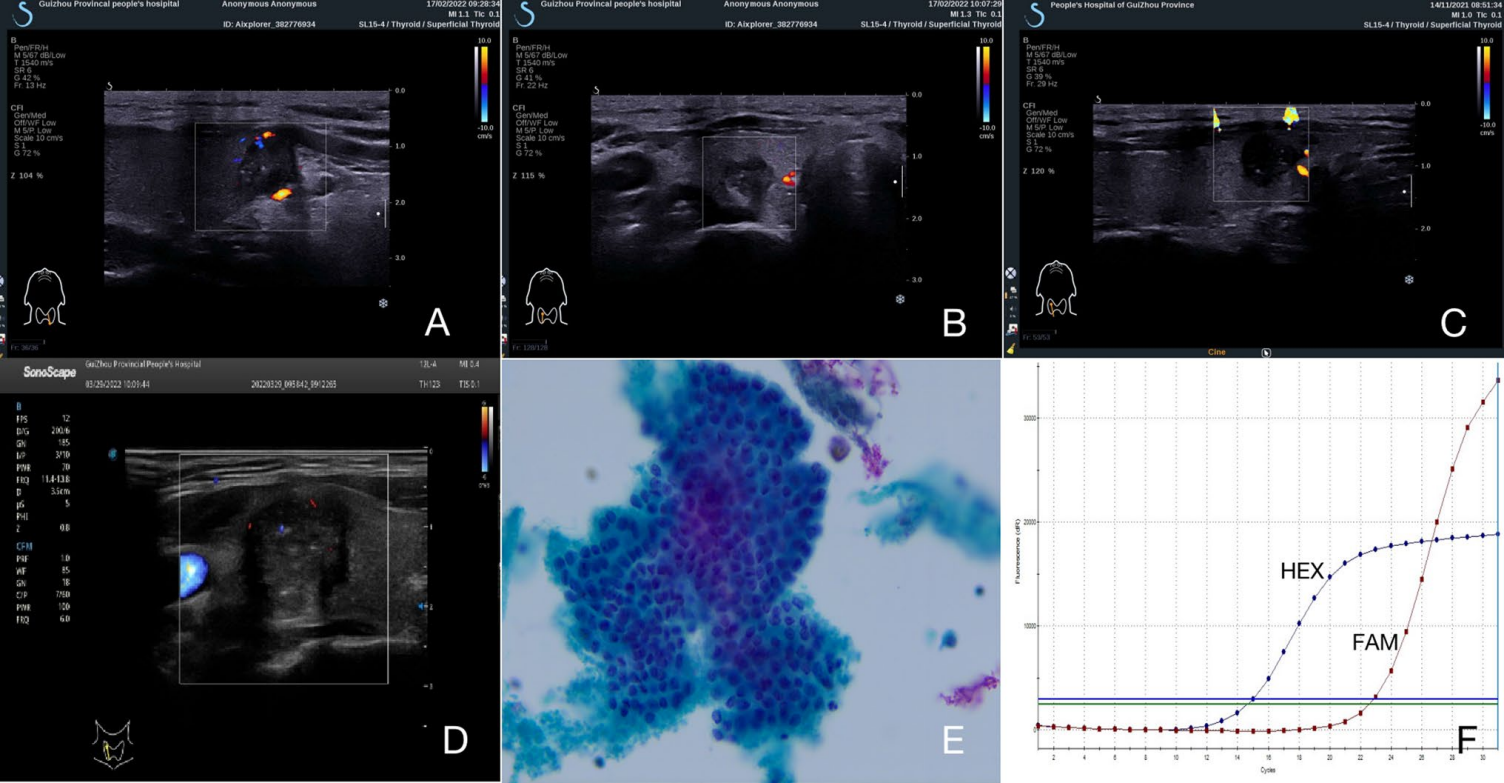
Four papillary thyroid carcinoma (PTC) patients with cervical central lymph-node metastasis (CLNM) and positive BRAF^V600E^ mutation. **A**–**D** The thyroid nodule had a low-echo, microcalcifications, capsule contact, and irregular morphology on US images. Color Doppler flow image (CDFI) showed a few punctate blood flow signals inside the nodule. **E** US-guided FNAB confirmed the diagnosis of PTC with cervical CLNM (papanicolaou stain, × 400). **F** Positive results of US-guided FNAB combined with BRAF^V600E^ mutation by PCR, with a mutation signal CT value of 20.8

**Fig. 3:**
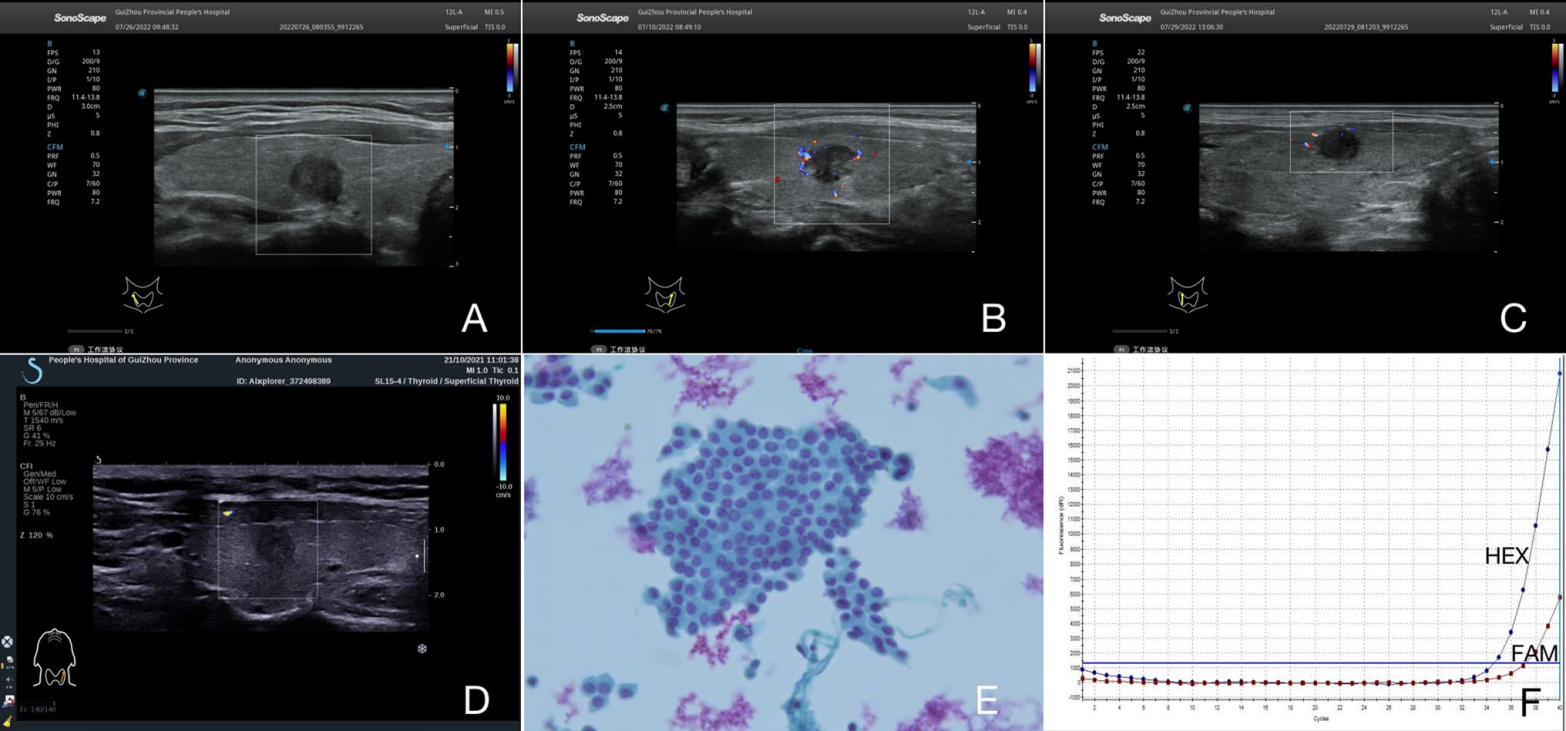
Four PTC patients with non-cervical CLNM and negative BRAF^V600E^ mutation. **A**–**D** The thyroid nodule had low-echo, non-microcalcifications, non-capsule contact, and clear boundary on US image. CDFI showed a lack of blood supply inside the nodule. **E** US-guided FNAB confirmed the diagnosis of PTC without cervical CLNM (papanicolaou stain, × 400). **F** Negative results of US-guided FNAB combined with BRAF^V600E^ mutation by PCR (no mutation)

After the strict inclusion, a total of 214 patients, including 151 (70.6%) females and 63 (29.4%) males, with median age of 42.5 years (range 21–69 years) were included for this research. Briefly, 123 (57.5%) were less than 45 years, and 91 (42.5%) were 45 years and older. 93 participants (43.5%) had cervical CLNM, whereas 121 (56.5%) did not. No distant metastases were found in our research. In 77 (36.0%) patients, the tumor size was ≤ 0.5 cm, 63 (29.4) patients had nodules between 0.5 and 1.0 cm, and the tumor size was > 1.0 cm in 74 (34.6%) patients. One hundred and seventy-five (81.8%) patients had unilateral nodule, whereas bilateral nodules were found in 39 (18.2%) patients. Suspicious US features with respect to taller than wide shape, capsule contact, microcalcifications, irregular morphology, unclear boundary, and rich blood flow were presented in 120 (56.1%), 91 (42.5%), 134 (62.6%), 173 (80.8), 108 (50.5%), and 78 (36.4%) of PTC samples, respectively. Typically, BRAF^V600E^ mutation was observed in 171 (79.9%) PTC patients, 46 (21.5%) participants were concomitant Hashimoto’s thyroiditis (HT), and 168 (78.5%) patients were without HT (Figs. 2, 3).

### The relationships between CLNM and the clinicopathological characteristics of PTC patients

**Fig. 4:**
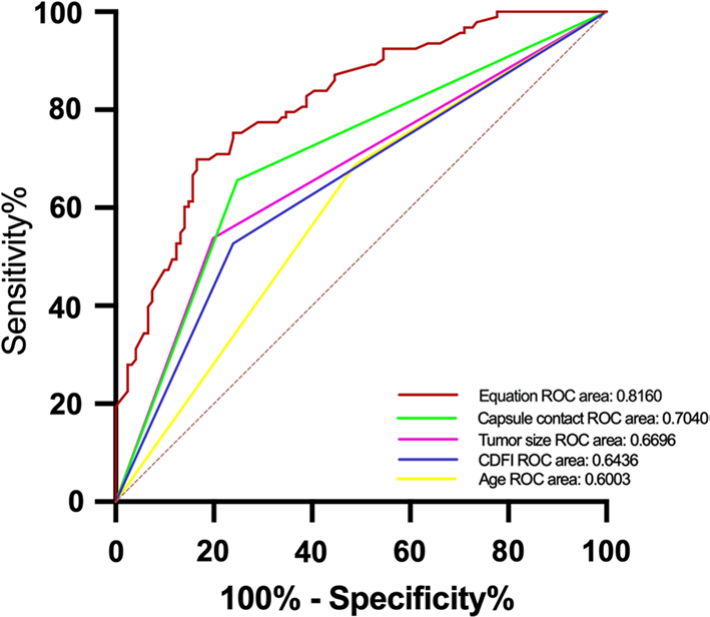
Receiver-operating characteristic (ROC) curves of PTC capsule contact [area under the ROC curve (AUROC) = 0.7040], tumor size (AUROC = 0.6696), CDFI (AUROC = 0.6436), age (AUROC = 0.6003), and equation (AUROC = 0.8160)] for the prediction of CLNM

As demonstrated in Table 1, participants less than 45 years of age have a higher frequency of cervical CLNM than those 45 years or older (52.0% vs 31.9%, *P = *0.003), and male patients had a higher risk of CLNM than female patients (54.0% vs 39.1%, *P = *0.045). Among US characterizes of PTC, maximum tumor size > 1.0 cm (*P < *0.001), tumor location with bilateral (*P = *0.004), capsule contact (*P < *0.001), microcalcifications (*P < *0.001), and rich blood flow (*P < *0.001) were remarkably correlated the presence of cervical CLNM. Other suspicious US characterizes, such as unclear boundary, irregular morphology, and solid component were not related to cervical CLNM in PTC (all P value > 0.05). In addition, non-concomitant HT was more inclined to be cervical CLNM (*P = *0.044). In papillary thyroid microcarcinoma (PTMC), using a cut-off value of 0.5 cm for the maximum tumor size, there was no statistically significant difference in the prediction of CLNM between the two groups (*P = *0.329). BRAF^V600E^ mutation was not significantly associated with CLNM positive in PTC patients (*P = *0.355). Moreover, independent risk factors were determined after multivariate analysis (Table 2). In our research, predictive risk factors, including age < 45 years (OR = 2.455, *P = *0.009), tumor size > 1.0 cm (OR = 2.586, *P = *0.010), capsule contact (OR = 3.208, *P = *0.001), and rich blood flow (OR = 2.225, *P = *0.022), turned out to be risk predictors for cervical CLNM in participants with cN0 PTC. We plotted the ROC curves, and the diagnostic value of the statistically significant factors was discriminative with area under the ROC curves of 0.6003 (95% 0.207–0.799), 0.6696 (95% 0.184–0.801), 0.7040 (95% 0.156–0.638), and 0.6436 (95% 0.227–0.894), respectively. The diagnostic value of the equation was discriminative with an area under the ROC curve of 0.8160 (95% 0.7596–0.8725) (Fig. 4).

**Table 1:** Basic clinicopathologic characteristics and US-guided FNAB features with cN0 PTC

Characteristics	Case *n* = 214(%)	Central CLNM (%)		*x*^2^	*p* value
		No	Yes		
Gender					
Female	151 (70.6)	92 (60.9)	59 (39.1)	4.014	0.045
Male	63 (29.4)	29 (46.0)	34 (54.0)		
Age, mean ± SD (years)	42.5 ± 10.97				
< 45	123 (57.5)	59 (48.0)	64 (52.0)	8.655	0.003
≧ 45	91 (42.5)	62 (68.1)	29 (31.9)		
Maximum tumor size (cm)					
≦ 0.5	77(36.0)	56(72.7)	21(27.3)	27.583	< 0.001
> 0.5, ≦ 1.0	63(29.4)	41(65.1)	22(34.9)		
> 1.0	74(35.6)	24(32.4)	50(67.6)		
Aspect ratio					
< 1	94 (43.9)	51 (54.3)	43 (45.7)	0.357	0.550
≧ 1	120 (56.1)	70 (58.3)	50 (41.7)		
Tumor location					
Unilateral	175(81.8)	107(61.1)	68(38.9)	8.272	0.004
Bilateral	39(18.2)	14(35.9)	25(64.1)		
Capsule contact					
Yes	91 (42.5)	30 (33.0)	61 (67.0)	35.811	< 0.001
No	123 (57.5)	91 (74.0)	32 (26.0)		
Microcalcifications					
Yes	134 (62.6)	63 (47.0)	71 (53.0)	13.241	< 0.001
No	80 (37.4)	58 (72.5)	22 (27.5)		
Boundary					
Clear	106 (49.5)	59 (55.7)	47 (44.3)	0.066	0.797
Unclear	108 (50.5)	62 (57.4)	46 (42.6)		
Morphology					
Regular	41 (19.2)	27 (65.9)	14 (34.1)	1.790	0.181
Irregular	173 (80.8)	94 (54.3)	79 (45.7)		
Component					
Solid	197 (92.1)	109 (55.3)	88 (44.7)	1.483	0.223
Cystic-solid	17 (7.9)	12 (70.6)	5 (29.4)		
BRAF^600E^ mutation					
Positive	171 (79.9)	94 (55.0)	77 (45.0)	0.855	0.355
Negative	43 (20.1)	27 (62.8)	16 (37.2)		
CDFI					
Poor	136 (63.6)	92 (67.6)	44 (32.4)	18.726	< 0.001
Rich	78 (36.4)	29 (37.2)	49 (62.8)		
HT					
Concomitant	46 (21.5)	32 (70.0)	14 (30.0)	4.044	0.044
Non-concomitant	168 (78.5)	89 (53.0)	79 (47.0)

**Table 2:** Multivariate logistic regression analysis of the relationship between predictors of PTC (significant by univariate analysis) and cervical CLNM

Independent variable	OR	95% CI		*P* value
		Lower	Upper	
Gender				0.070
Female	Reference	Reference		
Male	1.983	0.947	4.153	
Age				0.009
≧ 45	Reference	Reference		
< 45	2.455	1.251	4.820	
Maximum tumor size (cm)				0.010
≦ 1.0	Reference	Reference		
> 1.0	2.586	1.254	5.330	
Tumor location				0.113
Unilateral	Reference	Reference		
Bilateral	2.033	0.846	4.890	
Capsule contact				0.001
No	Reference	Reference		
Yes	3.208	1.634	6.297	
Microcalcifications				0.072
No	Reference	Reference		
Yes	1.903	0.945	3.833	
CDFI				0.022
Poor	Reference	Reference		
Rich	2.225	1.122	4.411	
HT				0.264
Concomitant	Reference	Reference		
Non-concomitant	1.610	0.698	3.711

### ***The relationships between the BRAF***^***V600E***^*** mutation and the clinicopathological characteristics of PTC patients***

The relationships between the BRAF^V600E^ mutation and the clinicopathological characteristics of the PTC patients are detailed in Table 3. BRAF^V600E^ mutation was detected in 214 patients with a positive frequency of 79.9% (171/214). Univariate analysis found that only capsule contact (*P = *0.001) was one of possible predictive factors of BRAF^V600E^ mutation status. No difference was found in cervical CLNM, maximum tumor size, tumor location, microcalcifications, CDFI, and HT.

**Table 3:** Correlation between clinicopathological characteristics and BRAF^V600E^ mutation in PTC

Characteristics	Case *n* = 214(%)	BRAF^V600E^ mutation (%)		*x*^2^	*P* value
		No	Yes		
Cervical CLNM					
Yes	93 (43.5)	16 (17.2)	77 (82.8)	0.855	0.355
No	121 (56.5)	27 (22.3)	94 (77.7)		
Maximum tumor size (cm)					
≦ 1.0	140 (65.4)	26 (18.6)	114 (81.4)	0.584	0.445
> 1.0	74 (34.6)	17 (23.0)	57 (77.0)		
Tumor location					
Unilateral	175(81.8)	34 19.4()	141 (80.6)	0.264	0.607
Bilateral	39(18.2)	9 (23.1)	30 (76.9)		
Capsule contact					
Yes	91 (42.5)	9 (9.9)	82 (90.1)	10.266	0.001
No	123 (57.5)	34 (27.6)	89 (72.4)		
Microcalcifications					
Yes	134 (62.6)	28 (20.9)	106 (79.1)	0.144	0.705
No	80 (37.4)	15 (18.8)	65 (81.2)		
CDFI					
Poor	136 (63.6)	30 (22.1)	106 (77.9)	0.898	0.343
Rich	78 (36.4)	13 (16.7)	65 (83.3)		
HT					
Concomitant	46 (21.5)	11 (23.9)	35 (76.1)	0.532	0.466
Non-concomitant	168 (78.5)	32 (19.0)	136 (81.0)		

## Discussion

Despite the favorable prognosis and high cure rate associated with most thyroid cancers, the incidence of cervical CLNM in PTC ranges from 20 to 90% according to the literature [14]. Inadequate preoperative staging by clinicians can lead to poor surgical outcomes and, in some cases, necessitate reoperation. A thorough preoperative staging evaluation can improve the selection of treatment plans and prognosis assessment, reduce the uncertainty caused by inaccurate preoperative staging, and significantly enhance the effectiveness of surgical treatment [15]. This is of great significance for improving clinical prognosis and reducing the rate of reoperation.

The preoperative diagnosis of CLNM is challenging due to anatomical factors, such as carotid pulse and potential small metastases. While preoperative ultrasound diagnosis is more sensitive for thyroid nodules, its sensitivity for preoperative diagnosis of cervical CLNM is as low as 12.1% [7]. About 60% of PTC patients have pathological evidence of cervical CLNM during their first surgery despite the absence of metastatic lesions before surgery. However, the determination of the surgical plan remains highly controversial. In 2020 [16], the American Association of Endocrine Surgeons released a new version of the “Guidelines for the Definitive Surgical Management of Thyroid Disease in Adults,” which does not recommend preventive neck lymph-node dissection for patients in T1, T2, and cN0 stages. Nevertheless, controversy remains over whether this conservative approach is appropriate for patients in China. Yang P et al. [17] believe that there is still insufficient evidence to recommend this surgical method as a routine surgical approach for patients in China. Some studies [8, 18] indicate that pCLND can prevent long-term metastasis and recurrence of thyroid cancer with high safety, even in the absence of infiltration or metastasis of lymph nodes during surgery on patients with PTC. In our study, 43.5% (93/214) of PTC patients who underwent pCLND were found to have cervical CLNM by postoperative histopathology results, suggesting that occult metastatic lymph nodes would have gone undetected if pCLND was not performed. The present study collected FNAB and BRAF molecular testing data from confirmed PTC cases and aimed to predict the occurrence of CLNM in PTC patients with cN0 through comprehensive analysis of US features and clinical indicators. The study also aimed to provide a reference for clinical decision-making regarding surgical approach.

FNAB remains a reliable diagnostic test for thyroid cancer. US-guided FNAB technology is widely used to improve diagnostic accuracy through cytologic or genomic examination [19]. Currently, the clinical basis for molecular diagnosis using FNAB is mostly derived from distinguishing between benign and malignant thyroid nodules in Class III and Class IV, with a particular focus on identifying benign nodules to prevent unnecessary surgery. Furthermore, the ability of BRAF detection in cytology is not inferior to that in pathological specimens after surgery. Multipoint and multidirectional US-guided FNAB is a new and superior puncture method compared to previous methods such as single-point and single-direction repeated puncture, and single-point and multidirectional puncture, especially for nodules less than 5 mm during percutaneous thyroid nodule biopsy (PTMC) procedures [20]. Multipoint and multidirectional US-FNAB can obtain cells from multiple locations, reducing the risk of obtaining invalid or false-negative specimens due to limited or suboptimal puncture sites, or bleeding caused by repeated puncture at a single point. This method improves the overall diagnostic yield. In our study, we used multipoint and multidirectional puncture combined with BRAFV600E mutation detection to determine the nature of thyroid nodules. This method demonstrated high diagnostic accuracy and sensitivity when compared to postoperative pathology.

This study conducted a univariate analysis to explore the relationship between cervical CLNM and clinical and US images in patients with PTC. Factors associated with cervical CLNM in PTC patients included male gender, age under 45 years, tumor size greater than 1.0 cm, nodules on both sides, contact with the thyroid capsule, presence of microcalcifications and rich blood flow, and absence of concomitant Hashimoto's thyroiditis. Initially, we used the age cutoff of 55 years based on the recent AJCC criteria[21] and found no significant correlation between age and cervical CLNM (*P = *0.503). However, upon reviewing the previous version, we revised the analysis to include individuals aged 45 years, which revealed a correlation between age and cervical CLNM consistent with the previous studies [22, 23]. This study may have collected a majority of cases around the age of 43 years. Multivariate logistic regression analysis suggests that advanced age is a protective factor for the occurrence of CLNM, which is consistent with the findings of other scholars [24]. Our study found that cervical CLNM is more common in male PTC patients (*P = *0.045). However, multivariate logistic regression analysis indicated that male gender is not an independent risk factor for CLNM. Other relevant studies [25] have suggested that male patients are associated with cervical CLNM, possibly due to the higher incidence of thyroid cancer in females. However, the relatively small number of male cases may have led to selection bias, and the varying inclusion and exclusion criteria in different studies indicate a need for further research and exploration of related factors.

Previous studies have demonstrated that suspicious US features, including a size greater than 1.0 cm, bilateral nodules, capsule contact, microcalcifications, and rich blood flow, as well as non-concomitant HT, are predictors of cervical CLNM in patients with PTC. These features indicate heavier tumor burden and invasiveness, and they are independent risk factors [26–29]. For PTMC, we discovered that LNM was not associated with the nodules' size when using a maximum tumor size reference of 0.5 cm. This result is consistent with Shi et al.'s research [24]. However, further research is necessary to validate this finding. After incorporating the aforementioned risk factors into multivariate logistic analysis, bilateral nodules, microcalcifications, and non-concomitant HT were no longer significantly associated with the occurrence of cervical CLNM. Conversely, patient age less than 45 years, size greater than 1.0 cm, capsule contact, and rich blood flow were independent predictors of cervical CLNM in cN0 PTC patients. Combining US-specific changes such as tumor size, capsule contact, and rich blood flow with patient age can improve the accuracy of CLNM prediction in PTC patients, with an area under the AUC curve of 0.816.

The use of molecular diagnostic testing has had a significant impact on the diagnosis and management of thyroid nodules and thyroid cancer [30]. Our retrospective study utilized preoperative FNAB specimens to detect BRAF mutations. We discovered that 79.9% (171/214) of patients with PTC exhibited the BRAF^V600E^ mutation. However, we did not find significant association between BRAF^V600E^ mutation and CLNM. As the decision to conduct BRAF^V600E^ mutation tests is at the discretion of surgeons, there may be a selection bias in the study. In addition, the BRAF^V600E^ mutation analyses were performed in a relatively small number of patients; therefore, the rate of BRAF^V600E^ mutation may be underestimated, leading to the absence of a significant correlation between BRAF^V600E^ mutation and aggressive factors. A larger multicenter study may be necessary [24]. The BRAF^V600E^ mutation has been identified as the most common genetic change in PTC. The mutations activate the RAS/RAF/mitogen-activated protein kinase pathway and cause malignant cell proliferation. The BRAF^V600E^ mutation is more commonly found in highly aggressive subtypes, such as tall cell PTMC, indicating a significant association between BRAF^V600E^ mutational status and poor tumor behavior [9]. In our study, BRAF^V600E^ was analyzed separately, indicating that capsule contact may be a risk factor for BRAF^V600E^ mutation; this also confirms the correlation between BRAF^V600E^ mutation and high invasiveness. Lim et al. [31] analyzed the BRAF^V600E^ mutational status of highly aggressive PTMC and discovered that the rate of BRAF^V600E^ mutation was higher in patients with capsule penetration compared to those without, which is similar to our conclusion. A meta-analysis [32] involving 9924 FNAB samples confirmed that the specificity and sensitivity of BRAF^V600E^ mutation in diagnosing PTC were 100% and 69%, respectively. However, there is currently a debate on whether BRAF^V600E^ mutations are related to CLNM in PTC patients. Some scholars' research suggests that the presence of BRAF^V600E^ mutations can significantly increase the probability of CLNM in PTC patients [33, 34]. However, other scholars' research indicates that the BRAFV600E mutation in LNM may not be related to the invasive characteristics of LNM in PTC [7, 9, 35–37]. Our study found that the incidence of CLNM in patients with positive BRAF^V600E^ mutation (77/171 = 45.03%) was higher than in patients with negative mutation (16/43 = 37.21%), suggesting that BRAF^V600E^ mutation may promote LNM of PTC.

Recently, predictive risk factor analysis based on clinical data has been utilized in various medical conditions. It has made significant progress. To address these concerns, this research collected and analyzed clinical data from cN0 PTC patients for retrospective analysis. Four independent risk factors were identified from numerous related factors, including age, maximum tumor size, capsule contact, and CDFI. Targeted neck lymph-node dissection was performed during surgery for precise treatment of occult CLNM.

Limitations of this study include the retrospective nature of the study, with all data collected from the same hospital, which may lead to bias. Additionally, US examination and analysis may be influenced by subjective factors, leading to errors in the judgment of cervical lymph nodes. To improve the applicability and clinical value, subsequent studies should conduct grouping studies on the patient's course of disease and conduct multicenter, large sample prospective studies.

## Conclusion

Our analysis identified that age under 45 years, size greater than 1.0 cm, capsule contact, and rich blood flow are significant predictors for cervical CLNM in cN0 PTC patients. Prior to establishing a definitive treatment plan for the patient, a range of analyses, including US, assessments of patient clinical characteristics, and US-guided FNAB, should be performed. As not all PTC (cN0) lesions are identical, the formulation of appropriate individualized therapeutic regimens is dependent on comprehensive analyses of all of these pertinent risk factors.

## Data Availability

The data that support the findings of this study are available on request from the corresponding author. The data are not publicly available due to privacy or ethical restrictions.
